# Tracking the Prevalence of FAS

**Published:** 1994

**Authors:** José F. Cordero, R. Louise Floyd, M. Louise Martin, Margarett Davis, Karen Hymbaugh

**Affiliations:** José F. Cordero, M.D., M.P.H., is assistant director for science; R. Louise Floyd, D.S.N., R.N., is chief of the Fetal Alcohol Prevention Section; M. Louise Martin, D.V.M., M.S., is chief of the Surveillance Unit; Margarett Davis, M.D., M.P.H., is medical epidemiologist; and Karen Hymbaugh, M.P.A., is behavioral scientist in the Division of Birth Defects and Developmental Disabilities, National Center for Environmental Health, Centers for Disease Control and Prevention, Atlanta, Georgia

## Abstract

Surveillance programs allow the tracking of the prevalence of a condition over time. Tracking the prevalence of FAS poses particular problems, however, as there is no “gold standard” of diagnosis. To evaluate the effectiveness of prevention efforts, surveillance techniques must be refined.

Fetal alcohol syndrome (FAS) is a birth defect that causes significant lifetime disabilities (Abel 1990). But unlike many other birth defects, FAS, which is caused by maternal alcohol abuse during pregnancy, is preventable (Abel 1990). In fact, prevention of FAS is a national health priority included in the *Healthy People 2000* objectives for health promotion and disease prevention (U.S. Department of Health and Human Services 1990 [USDHHS]). The specific health objective is to reduce the rate of FAS to no more than 1.2 cases per 10,000 live births by the year 2000.

Baseline data for this objective were derived from a national hospital-based epidemiologic surveillance program of birth defects—the Birth Defects Monitoring Program (BDMP) of the Centers for Disease Control and Prevention (CDC). Although the rate of 5.2 cases per 10,000 live births in 1992 seems to be an increase over the baseline rate of 2.2 cases per 10,000 (USDHHS 1990), it more likely represents improvements over recent years in recognition and reporting of FAS at birth.

In this article, we review the challenges of developing simple and efficient State-based and national epidemiologic surveillance that can track what progress is being made toward meeting the *Healthy People 2000* objective for FAS.

## Epidemiologic Surveillance

CDC defines epidemiologic surveillance as the ongoing systematic collection, analysis, and interpretation of health data that are essential to the planning, implementation, and evaluation of public health practice (Thacker et al. 1989). Such surveillance is closely integrated with timely dissemination of these data to anyone who requires this information. To monitor progress in meeting the FAS prevention objective, epidemiologic surveillance is needed to evaluate changes in the rate of FAS over time.

### Methodological Problems

#### Diagnosing FAS

Developing surveillance of FAS presents unique challenges. Because there is no simple, objective laboratory test for diagnosing FAS, diagnosis is based primarily on clinical definitions developed for the purpose of clinical practice and research (Sokol and Clarren 1989). To meet the clinical FAS case definition, the patient must exhibit symptoms in each of the following three categories: (1) prenatal or postnatal growth retardation; (2) central nervous system abnormalities; and (3) characteristic abnormal facial features (dysmorphology), including short palpebral fissures (eye openings), an elongated midface, a long and flattened philtrum (area between the nose and mouth), and a thin upper lip (see the photograph on p.13, in the article by Becker et al.). It is also helpful to determine if the patient was exposed prenatally to alcohol, but diagnosis can still be made if such information is unavailable (Sokol and Clarren 1989).

Applying these diagnostic criteria requires expertise in recognizing dysmorphic features. Moreover, the clinical features of a child with FAS may change with age (Streissguth et al. 1991; see the article by Streissguth, pp. 74–81). There is encouraging evidence that the clinical recognition and reporting of FAS is improving (CDC 1993*a*). However, such improvements may prove troublesome by clouding the true changes in the rate of FAS over time.

#### Collecting Data

CDC includes FAS in its two birth-defects surveillance programs. The first program is the BDMP, which relies on reported hospital discharge diagnoses of newborns that use the *International Classification of Diseases, Ninth Revision, Clinical Modification* (ICD–9–CM) (World Health Organization 1989). This program started monitoring FAS after 1979 when the ICD–9 introduced a code (760.71) that could be applied to the syndrome.

The second program is the Metropolitan Atlanta Congenital Defects Program (MACDP) (Lynberg et al. 1990), which started in 1968 and is the oldest active case-ascertainment birth-defects surveillance program in the United States. It monitors all births occurring in the five-county metropolitan Atlanta area—currently about 38,000 per year. The MACDP, unlike the BDMP, uses multiple sources to identify diagnoses of FAS in newborns and infants, including obstetrical, nursery, and pediatric logs. In addition, the MACDP identifies cases of FAS diagnosed through the first year of life, not solely at birth.

Because the BDMP, the MACDP, and other birth-defects surveillance programs examine only the medical records rather than the children directly, they rely on clinicians recognizing the condition and recording the diagnosis in the patient’s medical record. Further, the BDMP relies on the diagnosis of FAS during the newborn’s hospital admission and the recording of that diagnosis in the baby’s discharge record. The MACDP, on the other hand, detects diagnoses mentioned anywhere in a medical record, throughout the first year of a child’s life.

## Designing a Surveillance Program

Three important attributes of a successful surveillance program are sensitivity, predicted value positive, and representativeness (Klaucke 1992). These are defined and discussed below.

### Sensitivity of Surveillance

Sensitivity is a measure of how well the surveillance program detects cases of a condition. The greater the percentage of cases identified, the greater the chance to identify true changes in the rate of the condition over time.

#### Underdiagnosis

A case definition that only captures some of the FAS cases will decrease the surveillance program’s sensitivity. Sensitivity also will be decreased when surveillance is based only on clinical recognition of FAS, because the condition may sometimes be hard to discern. For example, the facial features that are characteristic of FAS can be missed by clinicians who may not be familiar with dysmorphology (Morse et al. 1992).

During a 6-year period, a separate longitudinal study examined prenatal exposure to alcohol in a large metropolitan hospital in Atlanta (Cordero et al. 1990). The study examined babies born at the hospital between 1980 and 1986, using a systematic checklist specific to the features of FAS. The concurrence of the study and the MACDP surveillance program allowed a comparison of their rates of diagnosis. Only 38 percent of the FAS cases diagnosed by the longitudinal study also were diagnosed by the MACDP (Cordero et al. 1990). This may indicate a lack of clinical recognition, a failure to record findings in the medical record, the use of variable criteria for the case definition, or a combination of all of these by the MACDP program.

#### Underreporting of Diagnosis

Another potential bias is that FAS may be recognized and recorded in medical records at different rates in different populations. About 75 percent of all recorded cases of FAS in the MACDP came from a large inner-city hospital that serves the poor and uninsured. Between 1975 and 1989, the inner-city hospital reported 88 FAS cases out of 98,000 births, whereas a comparable large suburban hospital reported 3 cases out of 72,000 births over the same period. Although it is possible that the rate of FAS in the two hospitals truly is different, lack of recognition of FAS, not recording the diagnosis, and bias in suspecting diagnosis should be evaluated as potential reasons for the difference.

In another study, which looked at the rate of diagnosis of FAS by pediatricians, a survey in Massachusetts found that among pediatricians who reported ever making a diagnosis of FAS, 9 percent also reported not recording the diagnosis in the medical record (Morse et al. 1992). The reasons for not recording the diagnosis were not explored. Regardless of whether the diagnosis is missed or not recorded, both events decrease the sensitivity of FAS surveillance and therefore will cause the underestimation of the rate of FAS.

#### Using a Limited Population

The child’s age when the diagnosis of FAS is made also may affect the sensitivity of the surveillance program. Surveillance of FAS based on newborn diagnosis will likely have a low sensitivity, because some affected newborns may have subtle facial abnormalities, inapparent central nervous system deficits, and normal birth weight (Abel and Sokol 1991). In addition, clinical, behavioral, and facial features may vary with the age of the child beyond the newborn period (Streissguth et al. 1985). In the MACDP, about 78 percent of the FAS cases were diagnosed during the newborn period (unpublished data). Surveillance programs that focus on the diagnosis of FAS at a specific age may tend to have lower sensitivity than others that cover all age groups.

In summary, several factors will affect the sensitivity of FAS surveillance. They include underdiagnosis, lack of recording of the diagnosis in the medical record, the possibility of stereotyping populations, inclusion of a limited population subset, and a restrictive case definition.

### Predictive Value Positive

A good surveillance program for FAS must identify the rate of true cases of FAS. Each case recorded using the surveillance case definition should have a high probability of being a true case of FAS. Predictive value positive (PVP) is the measure of how accurately cases are diagnosed given the nature of the case definition. FAS presents a particularly challenging situation for estimating PVP because there is no known “gold standard” for diagnosis. Even knowledgeable dysmorphologists often disagree about clinical diagnoses of FAS.

#### Low PVP

A case definition that allows inclusion of noncases will decrease the PVP of the surveillance. For example, if an FAS surveillance program uses a case definition of intrauterine growth retardation and a history of maternal alcohol abuse only but not the dysmorphic facial features, it is likely that many cases included under that definition may not meet the clinical criteria of FAS.

Clinical features in each of the three categories of the current case definition are not unique to FAS; they may result from many different causes. For example, the general definition of intrauterine growth retardation is weight and length below the 10th percentile for gestational age. Using this definition, about 10 percent of all newborns would meet this criterion, most of whom will not have FAS.

#### High PVP

Selecting a case definition that has a high PVP may decrease the sensitivity of the surveillance program. One can develop a definition for a condition that has such a high PVP that there is little doubt that each recorded case is a true case of the condition. Such a specific definition, however, also may miss many true cases, thereby greatly lowering the program’s sensitivity.

An example of this dilemma is a report of the sensitivity and PVP of isotretinoin embryopathy, or birth defects caused by maternal use of retinoic acid. The case definition with the highest PVP had a sensitivity of only about 16 percent (i.e., 84 percent of all true cases were missed) (Lynberg et al. 1990). However, the case definition avoided counting false cases. If the intent of the surveillance is to follow the trends of a condition, without trying to ascertain its incidence, such an approach may be adequate. In the situation of FAS, however, determining the incidence is quite important, given the current *Healthy People 2000* objective and the potential use of the surveillance as a registry that may track access of services to affected children.

### Representativeness

Representativeness is the ability of the surveillance program to reflect accurately the occurrence of the condition over time in the population of interest.

A major purpose of FAS surveillance on a national level is to gauge the trends in the U.S. population. Representativeness ensures that the picture painted by the surveillance program reflects the picture of the Nation. For example, using data from the BDMP, Chávez and colleagues (1988) found that the reported rate of FAS varies by race and ethnic group. If the surveillance is not population based, as with the case of the BDMP, and the racial and ethnic distribution does not reflect that of the general population, the findings may not be generalized except with appropriate adjustments.

The rate of FAS in a population is dependent on the rate of maternal alcohol abuse during pregnancy. If a specific sub-population included in the surveillance program has a rate of alcohol abuse during pregnancy different from that of the general population, the representativeness of the surveillance also will be affected. Stereotyping that affects the program’s definition or diagnosis of FAS also can affect representativeness.

## Estimating the Extent of the Problem

### The Estimates

**Figure 1:**
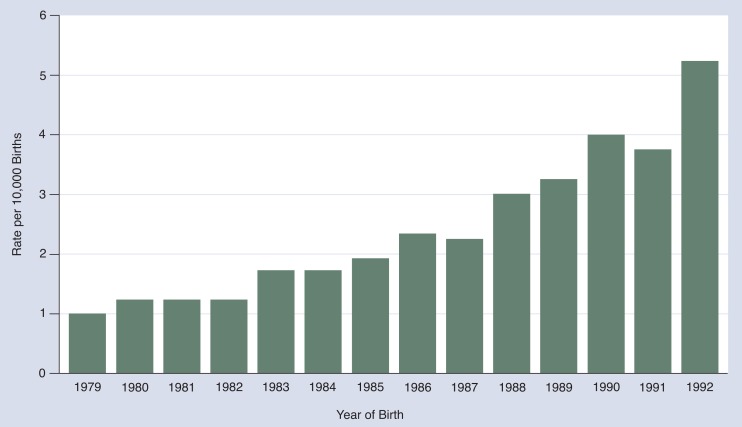
Reported incidence rate of fetal alcohol syndrome, by year of birth, from the Birth Defects Monitoring Program of the Centers for Disease Control and Prevention, 1979–1992.

The estimated birth prevalence of FAS among newborns identified through the BDMP increased from about 1 case per 10,000 live births in 1979 to 5.2 cases per 10,000 live births in 1992 (figure 1) (CDC 1993*a*). As mentioned above, the BDMP is based on doctors recording the ICD–9–CM FAS diagnosis code at discharge from the hospital of birth, and the program has not been evaluated in terms of its sensitivity and PVP. In 1992, the MACDP reported a rate of 3.3 cases per 10,000 live births. Both programs crudely estimate the average incidence of FAS for the reporting period 1979–1992 as 2 cases per 10,000 live births (Lynberg and Edmonds 1992). It is difficult to tell whether the reported increase during the last decade represents a true increase or the increased awareness and improved reporting among health care professionals. Interestingly, Serdula and colleagues (1991) reported that between 1985 and 1988, the percentage of women who drank during pregnancy actually *declined*. However, this decline was not evident for less educated women and women under age 25 years.

Abel and Sokol (1991)^1^ estimated the rate of FAS to be 3.3 cases per 10,000 live births. This estimate is based on the results of 15 prospective studies and 4 surveillance studies from Australia, New Zealand, Sweden, the United Kingdom, and the United States (Abel and Sokol 1991). None of these studies was population based.

### Limitations of the Estimates

Potential limitations of the CDC data sources include decreased sensitivity arising from decreased recognition of FAS in newborns (CDC 1993*a*), possible failure to incorporate the FAS diagnosis into the medical records (CDC 1993*b*), and the possible inappropriate use of the ICD–9–CM FAS code for reporting prenatal exposure to alcohol. The MACDP and the BDMP were designed primarily to monitor major birth defects in infants—to identify structural birth defects that are evident at birth or have significant clinical manifestations during the first year of life. They were not designed to track syndromes such as FAS that have few major birth defects.

In their estimate, Abel and Sokol (1991) did not use data that were population based, and they did not include minorities, Native Americans in particular. This omission may underestimate the rate of FAS. A recent survey of FAS among Native Alaskans reported an FAS rate of 21 cases per 10,000 live births (CDC 1993*b*).

## Improving FAS Surveillance

Given the limitations of current national FAS surveillance, researchers must improve it by systematically determining the strengths and shortcomings of existing surveillance, for example, through evaluation of the sensitivity, PVP, and representativeness of these existing programs. In addition to improving the national surveillance program, researchers should focus attention on State-based FAS surveillance. The proportion of hospitals participating in the BDMP is declining, and participation in some States is insufficient to estimate rates of FAS for these States.

The first task in improving surveillance is to develop a uniform case definition of FAS that optimally balances sensitivity with precision and can be used for surveillance. This should be followed by uniformity in application of the case definition. Also, the development of sensitive and specific screening instruments, including biologic markers, could contribute greatly to better epidemiologic data in this field. The second task is to ensure uniformity in methods of reporting data and in surveillance programs to receive the data. The combined effort of all concerned with the prevention of FAS will help make improved surveillance of FAS a reality.

## Footnotes

1 An earlier study of Abel and Sokol (1987) that surveyed 19 published epidemiologic studies worldwide reported an overall rate from all studies of 1.9 cases of FAS per 1,000 live births (i.e., 19 cases per 10,000 live births).
